# Extraskeletal Functions of Vitamin D

**DOI:** 10.1155/2015/294719

**Published:** 2015-05-11

**Authors:** Domenico Santoro, Katarina Sebekova, Daniel Teta, Luca De Nicola

**Affiliations:** ^1^Department of Clinical and Experimental Medicine, University of Messina, Viale Gazzi, 98123 Messina, Italy; ^2^Institute of Molecular BioMedicine, Medical Faculty, Comenius University, Sasinkova 4, 811 08 Bratislava, Slovakia; ^3^Department of Internal Medicine, Service of Nephrology, University Hospital (CHUV), Avenue du Bugnon 17, 1011 Lausanne, Switzerland; ^4^Nephrology, Med School, Second University of Naples, Piazza Miraglia, 80138 Naples, Italy

The recent identification of an expanded role for vitamin D action, beyond its traditional actions in mineral metabolism and the novel investigational approaches as well, has opened new therapeutic avenues for both the clinician at bedside and the scientist. This is the rationale of dedicating a special issue on this hot topic.

A new spectrum of vitamin D biological activities including important aspects on cellular proliferation, differentiation, and the immune system has been recently identified. Relevant is also the interaction of vitamin D with other kidney hormones such as renin and erythropoietin; this topic is specifically addressed by this paper. Indeed, the administration of vitamin D analogues has been associated with an improvement in anaemia and reduction in erythropoiesis-stimulating agents (ESA) requirements. Furthermore, vitamin D deficiency could contribute to the inappropriately activated or unsuppressed renin-angiotensin-aldosterone system (RAAS) in pathologic conditions.

Experimental data show that vitamin D may also interfere with the compensatory increase in renin synthesis occurring during chronic administration of anti-RAAS agents. In this context, use of vitamin D may be therefore suggested in patients treated with anti-RAAS but not reaching the safe threshold level of proteinuria. As further support to the hypothesis on the use of vitamin D analogues as antiproteinuric agents in proteinuric chronic kidney disease (CKD) are the data evidencing its anti-inflammatory effects. In this special issue, J. Egido et al. provided novel insights into the association between vitamin D deficiency and the activation of the RAAS, which is critical if one considers that local activation of this latter system mainly contributes to inflammation and tissue damage in renal diseases. Based on studies in human kidney proximal tubular cells, the authors propose a novel signalling pathway explaining, at least in part, the anti-inflammatory effects of paricalcitol in chronic kidney disease (CKD). Indeed, they found that the anti-inflammatory actions of paricalcitol may depend on the inhibition of TGF-*α*/ADAM17/EGFR pathway stimulated by aldosterone.

I. Guessous reviewed the role of vitamin D, as a marker of health status rather than a predictor of health outcomes. He analyzed the potential impact of currently ongoing randomized clinical trials (RCTs). The role of vitamin D in skeletal complications is also questioned, since, according to recent studies, vitamin D might not be as essential as previously thought for maintaining bone health.

Observational data support a link between vitamin D status and cardiovascular diseases, and vitamin D deficiency can thus be considered a cardiovascular risk marker. Regarding this issue, O. Marginean and I. Mozos discussed the role of vitamin D in blood pressure control, left ventricular hypertrophy, atrial fibrillation, the metabolic syndrome, and peripheral artery disease. Genotyping for vitamin D receptor variants could help to distinguish patients at risk of developing cardiovascular disease.

In a compelling review, P. Olivier et al. first describe the trophic effect of vitamin D in the skeletal muscle. Hypovitaminosis D is consistently associated with decrease in muscle function and performance and increase in disability. Data from randomized controlled trials and meta-analysis showed that vitamin D supplementation improves muscle strength, gait, and the risk of falls in different settings, especially in the elderly frail patient.

G. Ferlazzo et al. review data from clinical and basic research data on the role of vitamin D in inflammatory bowel disease (IBD) with regard to immune regulation, VDR (vitamin D receptor) polymorphism, disease activity and severity, and outcomes. Despite the growing evidence suggesting a role for vitamin D deficiency in the development of IBD, the exact role of vitamin D in IBD is still unclear and merits further investigation.

The article of P. Mansueto et al. focuses on the most recent epidemiological and experimental data looking at relationships between vitamin D and HIV infection. Clinical implications and potential benefits of vitamin D supplementation, in this particular setting, are explored in this comprehensive review.

Two papers in this issue discuss vitamin D in relation with gynecological and obstetrics diseases. O. Triolo et al. reviewed in detail the current knowledge of the association between VDR-mediated signalling pathways and vitamin D levels in polycystic ovary syndrome, endometriosis, and ovarian and breast cancer as well as in pathologies related to the maternal-fetal unit, such as preeclampsia, gestational diabetes mellitus (GDM), infertility, and in vitro fertilization. Pleskacova et al. compared midgestation and early postpartum vitamin D status in women with GDM versus controls. They showed that both groups present similarly low 25(OH)D levels and overall high prevalence of vitamin D deficiency. However, women with GDM history presented significantly lower 25(OH)D levels and higher prevalence of 25(OH)D deficiency postpartum. The above manuscripts suggest that, despite convincing associations between hypovitaminosis D and gynecological/obstetric diseases, a causal relationship needs to be confirmed in future investigations aimed at clarifying the mechanisms linking vitamin D metabolism and hormonal/metabolic pathways involved in these diseases.

The study by Kalousová et al. readdressed the important issue of vitamin D status in nondialysis and dialysis CKD patient. In particular, they studied the relationship between vitamin D concentration in plasma and vitamin D binding protein (VDBP). The authors confirmed that plasma levels of vitamin D are decreased in CKD, especially in dialysis patients. More importantly, they found that VDBP does not play a role in vitamin D deficiency because of enhanced production as compared with healthy controls that compensates urinary losses of this protein.

It is known that intracellular calcium concentration significantly increases in peripheral blood mononuclear cells (PBMCs) of CKD patients, possibly impairing the regulatory mechanisms maintaining cellular calcium homeostasis. L. Sikurova et al. evaluated in early CKD patients with vitamin D deficiency if treatment with cholecalciferol for 6 months may restore these abnormalities. They showed that vitamin D3 supplementation had a beneficial effect on disturbed cell calcium homeostasis in these patients with early CKD.

The study of K. Šebeková et al. addressed the question whether an excessive accumulation of advanced glycation end products (AGEs) in the skin of diabetic patients interferes with dermal vitamin D3 formation and whether hypovitaminosis D is associated with an increased formation and toxicity of AGEs. They showed that hypovitaminosis D is not associated either with enhanced AGE accumulation or with markers of inflammation. These data suggest that hypovitaminosis D seems to be of limited importance for the development of microinflammation and accumulation of AGEs.

As summarized here, the research field on the extraskeletal effects of vitamin D is continuously growing. In this special issue, we tried to give a glimpse, although somehow critical, of the most interesting studies in this topic. Clinical studies are however still needed to correctly translate in patients the results obtained in labs.

*Domenico Santoro* *Katarina Sebekova* *Daniel Teta* *Luca De Nicola*

